# Combined application of microbial inoculant and kelp-soaking wastewater promotes wheat seedlings growth and improves structural diversity of rhizosphere microbial community

**DOI:** 10.1038/s41598-023-48195-1

**Published:** 2023-11-24

**Authors:** Xin Song, Rui Zheng, Yue Liu, Zhaoyang Liu, Jian Yu, Jintai Li, Pengcheng Zhang, Qixiong Gao, Huying Li, Chaohui Li, Xunli Liu

**Affiliations:** 1 College of Forestry, Shandong Agriculture University, No. 61, Daizong Street, Taian, 271018 Shandong China; 2 Key Laboratory of National Forestry and Grassland Administration on Silviculture of the Lower Yellow River, Shandong Agricultural University, Taian, China; 3 Shandong Nongda Fertilizer Technology Co. Ltd, Taian, Shandong China

**Keywords:** Biological techniques, Ecology

## Abstract

Industrial processing of kelp generates large amounts of kelp-soaking wastewater (KSW), which contains a large amount of nutrient-containing substances. The plant growth-promoting effect might be further improved by combined application of growth-promoting bacteria and the nutrient-containing KSW. Here, a greenhouse experiment was conducted to determine the effect of the mixture of KSW and *Bacillus methylotrophicus* M4-1 (MS) vs. KSW alone (SE) on wheat seedlings, soil properties and the microbial community structure in wheat rhizosphere soil. The available potassium, available nitrogen, organic matter content and urease activity of MS soil as well as the available potassium of the SE soil were significantly different (*p* < 0.05) from those of the CK with water only added, increased by 39.51%, 36.25%, 41.61%, 80.56% and 32.99%, respectively. The dry and fresh weight of wheat seedlings from MS plants increased by 166.17% and 50.62%, respectively, while plant height increased by 16.99%, compared with CK. Moreover, the abundance and diversity of fungi in the wheat rhizosphere soil were significantly increased (*p* < 0.05), the relative abundance of *Ascomycetes* and *Fusarium* spp. decreased, while the relative abundance of *Bacillus* and *Mortierella* increased. Collectively, the combination of KSW and the plant growth-promoting strain M4-1 can promote wheat seedlings growth and improve the microecology of rhizosphere microorganisms, thereby solving the problems of resource waste and environmental pollution, ultimately turning waste into economic gain.

## Introduction

Kelp is a cheap and abundant resource in coastal agricultural areas, with a great potential for commercial development^1,2^. It is mainly used for the production of alginates, functional sugar alcohols, biomedical materials, marine cosmetics, functional food ingredients, and marine microbial fertilizers. However, a large amount of kelp-soaking wastewater (KSW) is generated during kelp processing. For example, Qingdao Mingyue Company (Qingdao, Shandong) processes 7300 tons of dried kelp, 15,000 tons of fresh kelp, and 3200 tons of salted kelp every year, yielding more than one million cubic meters of wastewater in the process. KSW mainly contains organic substances, such as polysaccharides, cellulose, proteins, and pigments, as well as large amounts of inorganic salts. Discharging wastewater without treatment would lead to a waste of resources and affects aquatic resources and aquaculture, causing harm to the marine environment and deterioration of seawater quality and even endangering human health^3–5^. At present, in the production and processing of kelp products, most manufacturers use simple sedimentation, floating, or centrifugal separation to discharge kelp wastewater after primary treatment, which is time-consuming and can negatively impact the environment. Therefore, rational utilization of KSW is of interest to both economy and environmental protection.

Research into the use of various commercial and experimental seaweed biostimulants to improve crop yields and relieve abiotic stress has intensified in recent years^6,7^. Bioactive substances extracted from seaweed are often used as fertilizers for agricultural and horticultural crops, to improve their yield and quality and reduce negative environmental impact^8^. Seaweed extract is rich in betaine, polysaccharides, phenolic compounds, plant hormones, mannitol, and a variety of inorganic salts^2,9,10^, while KSW contains large amounts of active substances, such as mannitol, betaine, fucoidan, and inorganic salts. Considering their similar composition, the application of KSW can be rationalized by drawing on the application of seaweed extract. Seaweed extract can be applied near the plant root or as a foliar spray to promote growth. Ashour et al. studied the effects of the commercially available liquid seaweed extract True-Alga-Max (TAM^®^) as a plant growth stimulant on the nutrition and antioxidant activities of pepper after foliar spraying application^7^. They showed that the yield of pepper increased significantly upon TAM^®^ treatment, especially when 0.5% (V/V) TAM was used, with a maximum yield of 4.23 kg m^−2^ and a significant production of active biological molecules, such as chlorophyll, ascorbic acid, phenolic compounds, flavonoids, and total nutrients. Furthermore, Yao et al.^11^ treated greenhouse tomatoes with seaweed extract as a water-soluble fertilizer under irrigation. They showed that the application of seaweed extract (SES) significantly increased tomato yield, by 4.6–6.9%, compared to the control. This finding was attributed to the increased photosynthetic capacity of tomato leaf. The application of SES also significantly increased tomato hardness and reduced the ripening time. According to other studies, seaweed extract increases plant yield as well as pest and disease resistance by increasing the activity of many of the plant’s own enzymes^12^, improves plant growth by increasing the production of plant secondary metabolites^13,14^, and protects the plant from bacterial pathogens by activating their own natural immune response^15^. These effects stem from a direct impact on the plant physiological and biochemical processes directly related to nutrient uptake and plant growth and from the effect on the physical, chemical, and biological properties of the soil, indirectly promoting plant growth. Moreover, the application of seaweed extract helps soil bacteria to recover from the negative effects of drought by supporting the rebuilding of soil microbial populations, especially species with important functions in soil nutrient transformation and cycling^16^. The indirect probiotic effect of seaweed extracts on plants improves soil physicochemical properties and enhances the biological activity of various biological enzymes in the soil, in addition to improving the structural composition of the soil microbial community^17^. Polysaccharides present in seaweed extract contribute to soil gel formation, water retention, and soil aeration, and polyanionic compounds contribute to cation fixation and exchange and are important for the fixation of heavy metals and soil remediation^18^. These findings inform rational utilization of KSW.

In addition to the above-mentioned bioactive substances that promote plant growth, some beneficial bacteria can also promote plant growth, such as Plant growth-promoting rhizobacteria (PGPR). PGPR are beneficial microorganisms that colonize plant rhizosphere, improve plant tolerance to biotic and abiotic stresses^19,20^. Common PGPR are bacteria from the genera *Pseudomonas*, *Bacillus*, *Agrobacterium*, *Erwinia*, *Flavobacterium*, *Pasteuria*, *Serratia*, and *Enterobacter*^21,22^. In general, PGPR promote plant growth directly, by increasing nutrient acquisition (nitrogen, phosphorus, potassium, and essential minerals) and regulating plant hormone levels or, indirectly, by inhibiting various pathogens and acting as biocontrol agents^23,24^.

When used as a microbial biofertilizer applied to the seed or soil, PGPR promote the growth and yield of a variety of crops. The application of PGPR in plants has been extensively studied. For example, He et al.^25^ reported on a PGPR strain that promotes the growth and root development of ryegrass by regulating plant hormone secretion. The strain also enhanced drought tolerance of ryegrass by improving the activity of antioxidant enzymes, regulating abscisic acid signaling, and maintaining plant growth. Further, Nawaz et al.^26^ showed that under saline-alkaline conditions, PGPR can be used as potential bioinoculants to augment the growth and yield of wheat by modulating morpho-physiological and biochemical attributes of both salt-tolerant and salt-sensitive plant varieties. Ji et al.^27^ also showed that, inoculation with PGPR strain JC-K3 stimulated the accumulation of wheat biomass and increased the plant-soluble sugar and chlorophyll content, decreased the Na absorption in shoot and leaf, and improved wheat tolerance to salt stress. As shown by other studies, PGPR enhance plant abiotic stress tolerance via a variety of complex mechanisms^28,29^. During growth, plants are affected by abiotic stresses and also frequently experience biological stresses. PGPR protect plants from pathogen invasion by producing antibiotics^30^, plant hormones^31,32^, and inhibiting pathogen growth^33^, or by activating the plant’s own defense function to resist biological invasion^34^.

In addition to using them as the sole treatment, PGPR can be combined with other plant growth-promoting substances for an enhanced promotion of plant growth. For instance, combining PGPR with chemical fertilizers improves fertilizer efficiency and plant growth stimulation. As reported by Paungfoo-Lonhienne et al.^35^, supplementing the soil with mixed nitrogen sources (inorganic and organic fertilizers) in the presence of PGPR significantly increases the shoot and root biomass of kikuyu grass, by 48% and 45%, respectively, compared with the control group. Analysis of the mineral nitrogen composition of leachates collected in week 3 to assess whether the combination of organic fertilizer with PGPR reduced nitrogen loss revealed that the addition of PGPR reduced the concentration of soil mineral nitrogen (mainly in the form of NO_3_). Combined with vigorous plant growth, PGPR also enhanced the acquisition and assimilation of nitrogen, improved the utilization efficiency of fertilizer nitrogen, and reduced the potential of environmental risk related to nitrogen leaching.

Prior to the advent of high-throughput sequencing, studies of microbial communities focused on culturable microorganisms or high-abundance taxa, and such methods were limited^36^. High-throughput sequencing technology can revolutionize our ability to characterize microbial diversity by enabling the investigation of community composition at a much greater phylogenetic resolution than ever before^37,38^. In this study, we investigated the effect of a simultaneous application of a growth-promoting bacterium, *B. methylotrophicus* M4-1, and KSW on wheat seedlings growth. We also investigated the effect of the simultaneous application on the soil physicochemical properties and on microbial diversity and community structure in the rhizosphere soil of wheat, using next-generation Illumina sequencing.

## Results

### Effects of KSW with or without PGPR M4-1 strain application on wheat seedlings growth

**Figure 1:**
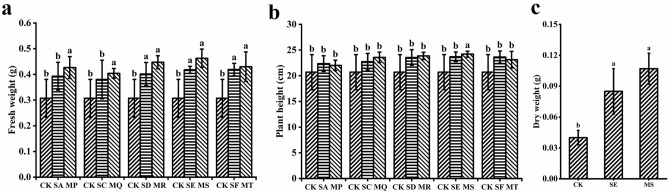
Effect of KSW, the mixture of KSW and M4-1 suspension on wheat seedlings. (**a**) Fresh weight, (**b**) Plant height, (**c**) Dry weight. Values are means ± SD (n = 6). Different letters indicate significant differences (*p* < 0.05) and the same letters indicate no significant differences (*p* > 0.05).

The effects of five different dilutions of kelp-soaked wastewater and the mixture of kelp-soaked wastewater at different dilutions with a suspension of the growth-promoting *B. methylotrophicus* M4-1 on wheat seedlings growth were investigated in a pot experiment. The results are shown in Fig. 1. The results showed that different dilutions of KSW had different growth-promoting effects on wheat seedlings. Among all the treatment groups, the mixture of kelp-soaked wastewater at 80 dilutions with a suspension of the growth-promoting bacterium *B. methylotrophicus* M4-1 (MS) had the most significant promotion effect on wheat seedlings, and the group of MS had the highest values of wheat seedlings fresh weight and plant height. As shown in Fig. 1a,b, the fresh weight of wheat seedlings increased by 50.62% and plant height increased by 16.99%, respectively.

As shown in Fig. 1, the dry weight, fresh weight, and plant height of wheat seedlings in SE and MS groups were higher than those in the control group (CK). The dry weight of wheat seedlings in the SE and MS groups were significantly higher than that in the control group (*p* < 0.05), and increased by 111.44% and 166.17% respectively, compared with the control (*p* < 0.05). The fresh weight of a single wheat seedlings in the SE group was 0.41 g and that in the MS group was 0.46 g, while the fresh weight of a single wheat seedlings in the CK group was only 0.3 g. The fresh weight of wheat seedlings in the MS group was significantly higher than that in the control group (*p* < 0.05). Measurement of the plant height of wheat revealed that both SE and MS treatments improved the plant height, to a certain extent. The plant height of wheat seedlings in the SE and MS groups increased by 14.45% and 16.99% respectively, compared with the control. In conclusion, both SE and MS treatments significantly promoted the growth of wheat seedlings; however, the growth-promoting effect of the latter was more pronounced than that of the former.

### Physical and chemical properties of soil in the SE and MS groups

**Figure 2:**
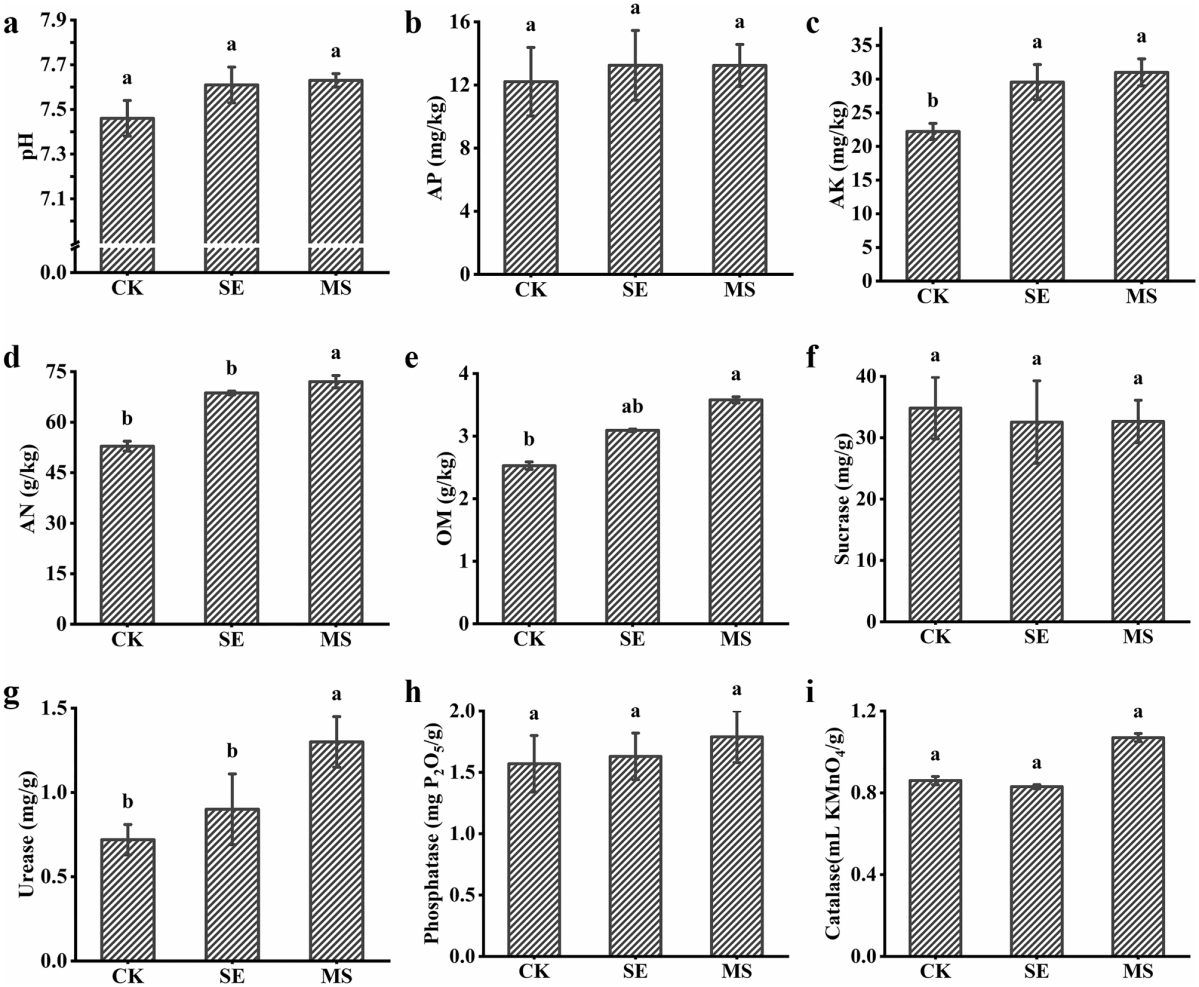
Effect of KSW, the mixture of KSW and M4-1 on soil physicochemical properties. (**a**) pH, (**b**) Available phosphorus, AP. (**c**) Available potassium, AK. (**d**) available nitrogen, AN. (**e**) Organic matter, OM. (**f**) Sucrase, (**g**) Urease, (**h**) Phosphatase, (**i**) Catalase. Values are means ± SD (n = 3).

Both SE and MS treatments affected the physical and chemical properties of the soil. In the current study, the available phosphorus, potassium and nitrogen level, organic matter content, and soil pH were evaluated as the soil fertility indices. The experiment revealed (Fig. 2) that the MS treatment significantly increased the available potassium content by 39.51%, increased the available nitrogen content by 36.25%, increased the organic matter content by 41.61%, and increased the urease content by 80.56% (*p* < 0.05). The SE treatment significantly increased the available potassium content by 32.99% (*p* < 0.05). The pH, available phosphorus, available nitrogen, organic matter, urease and phosphatase activity in the SE group were increased but not significantly different from those of the control group.

### Microbial community structure and diversity in wheat seedlings rhizosphere after SE and MS treatments

#### Assessment of microbial abundance and diversity

The diversity of wheat seedlings rhizosphere microbial community structure in the CK, SE, and MS rhizosphere soil samples was analyzed. The sequences were clustered using UPARSE algorithm at 97% similarity level. Finally, 3756 bacterial operational taxonomic units (OTUs) and 1411 fungal OTUs were obtained. Microbial diversity increased with the increase in sequencing depth, and all the curves leveled off, indicating that the sequencing became saturated and reflected species information for most microorganisms in the samples and that the sequencing data quality was sufficient for the ensuing analysis (Fig. S1).

**Figure 3:**
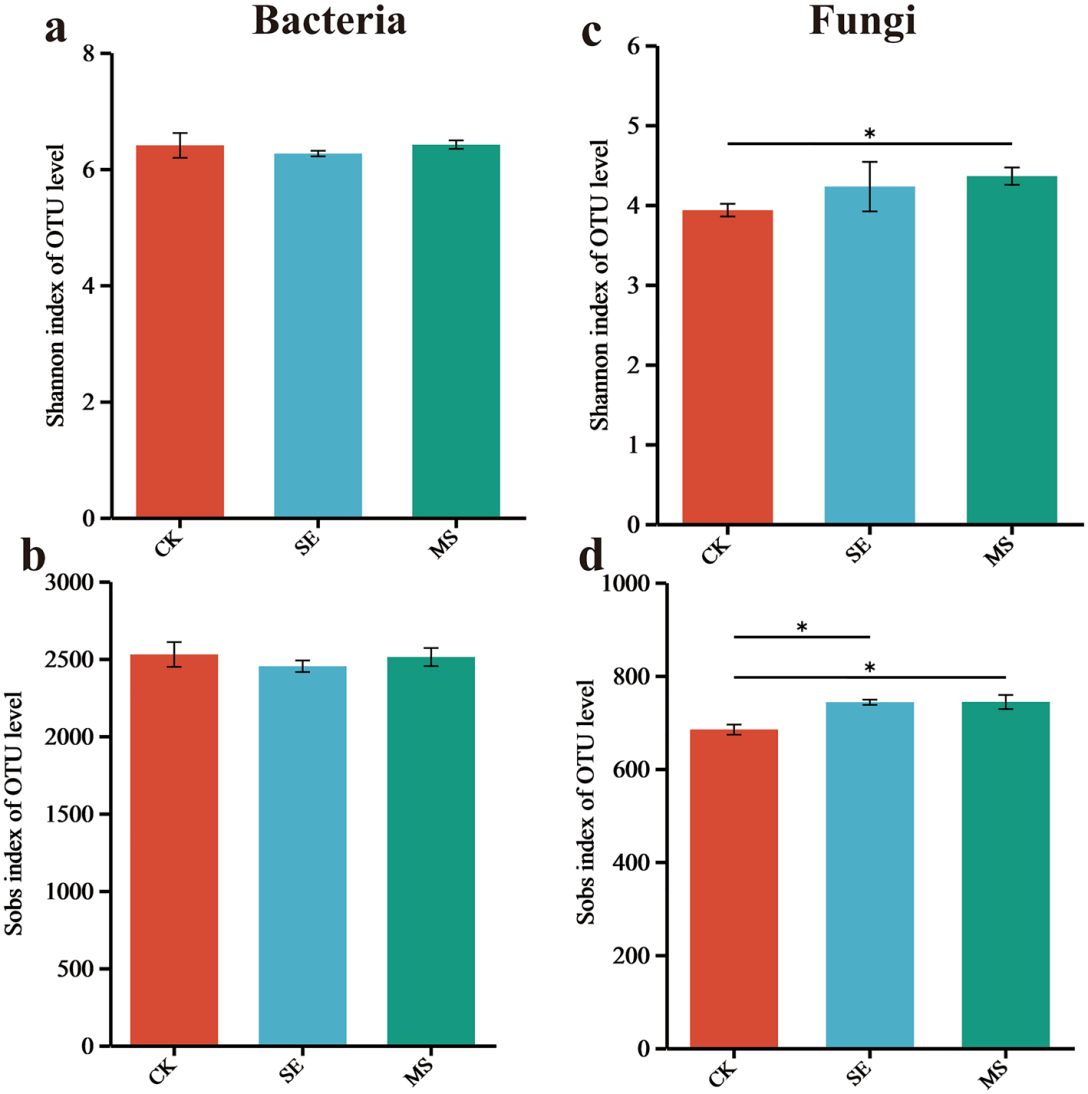
Shannon (**a**,**c**) and Sobs (**b**,**d**) diversity indices of bacteria and fungus communities in different treatment, *0.01 < p ≤ 0.05.

Shannon index reflect the community diversity in a sample. As shown in Fig. 3c, the diversity of fungal community in MS samples was significantly higher (*p* < 0.05) than that in CK samples, but with no significant effect on the diversity of the bacterial community. In SE samples, the diversity of the fungal community increased and the diversity of the bacterial community decreased, but neither change was significant. Sobs index reflect the community abundance in a sample. The bacterial community abundance in SE and MS samples were reduced compared to that in CK samples, the change was not significant (Fig. 3b). The fungal community abundance in SE and MS samples were significantly higher (0.01 < *p* < 0.05) than that in CK samples (Fig. 3d). These results suggest that the addition of kelp-soaked wastewater and microbial inoculants can change the microbial community structure in wheat rhizosphere soil to different extent. The principal coordinate analysis (PCoA) based on Bray–Curtis distance algorithm also confirms this conclusion (Fig. S2).

#### Bacterial and fungal community composition in SE and MS samples

**Figure 4:**
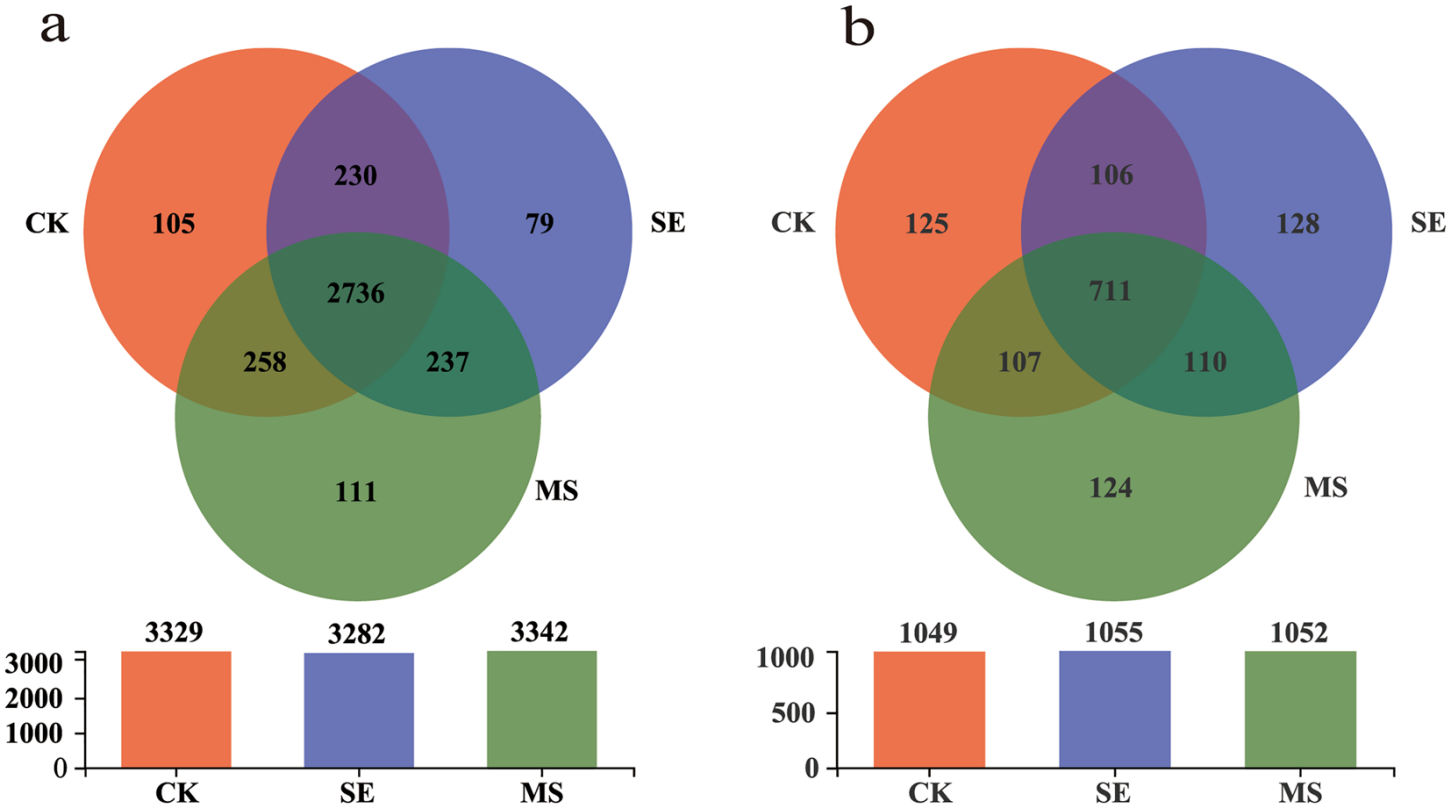
Venn diagram constructed at different OTUs levels of (**a**) bacteria and (**b**) fungus.

Venn diagram was used to visualize the similarity and overlap of species in the compared samples. Figure 4 revealed that the two treatment groups and the control group contained 9953 bacterial OTUs, with 105 OTUs specific to CK samples, 79 OTUs specific to SE samples, and 111 OTUs specific to MS samples. Collectively, all the sample types contained 3156 fungal OTUs, of which 125 were specific to CK samples, 128 were specific to SE samples, and 124 were specific to MS samples. Furthermore, 2736 bacterial OTUs and 711 fungal OTUs were shared among the sample types.

**Figure 5:**
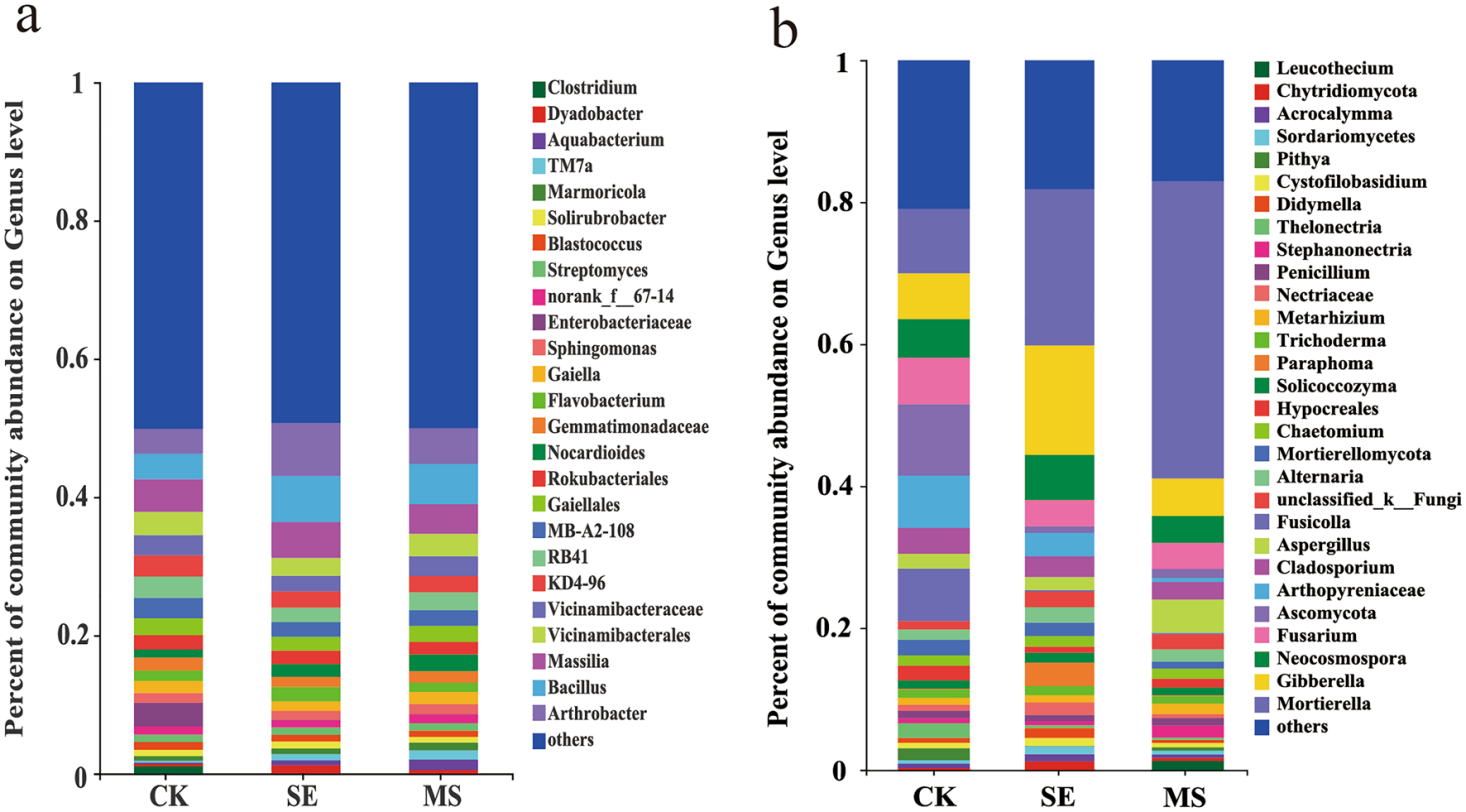
Composition and relative abundance of bacteria (**a**) and fungus (**b**) in different samples at the genus level.

Next, community histogram was used to visualize the community composition and relative abundance upon different treatments at different taxonomic levels based on the results of taxonomic analysis. Analysis of the bacterial community composition (Fig. 5a) revealed that the mixture of kelp-soaked wastewater and M4-1 bacterial suspension (MS) significantly increased the relative abundance of *Arthrobacter*, *Bacillus*, *Nocardioides*, and *Marmoriocia* in wheat rhizosphere soil. The relative abundance of *Enterobacterium* in wheat rhizosphere soil was significantly reduced in these samples. The analysis of fungal community composition at the genus level (Fig. 5b) revealed that the mixture of kelp-soaked wastewater and M4-1 bacterial suspension (MS) significantly increased the relative abundance of *Mortierella*, *Aspergillus*, and *Stephanonectria* in wheat rhizosphere soil. The relative abundance of pathogenic fungi, such as *Fusarium*, *Fusicolla*, *Cladosporium*, *Neocosmospora*, and *Gibberella*, in wheat rhizosphere soils was also substantially reduced.

**Figure 6:**
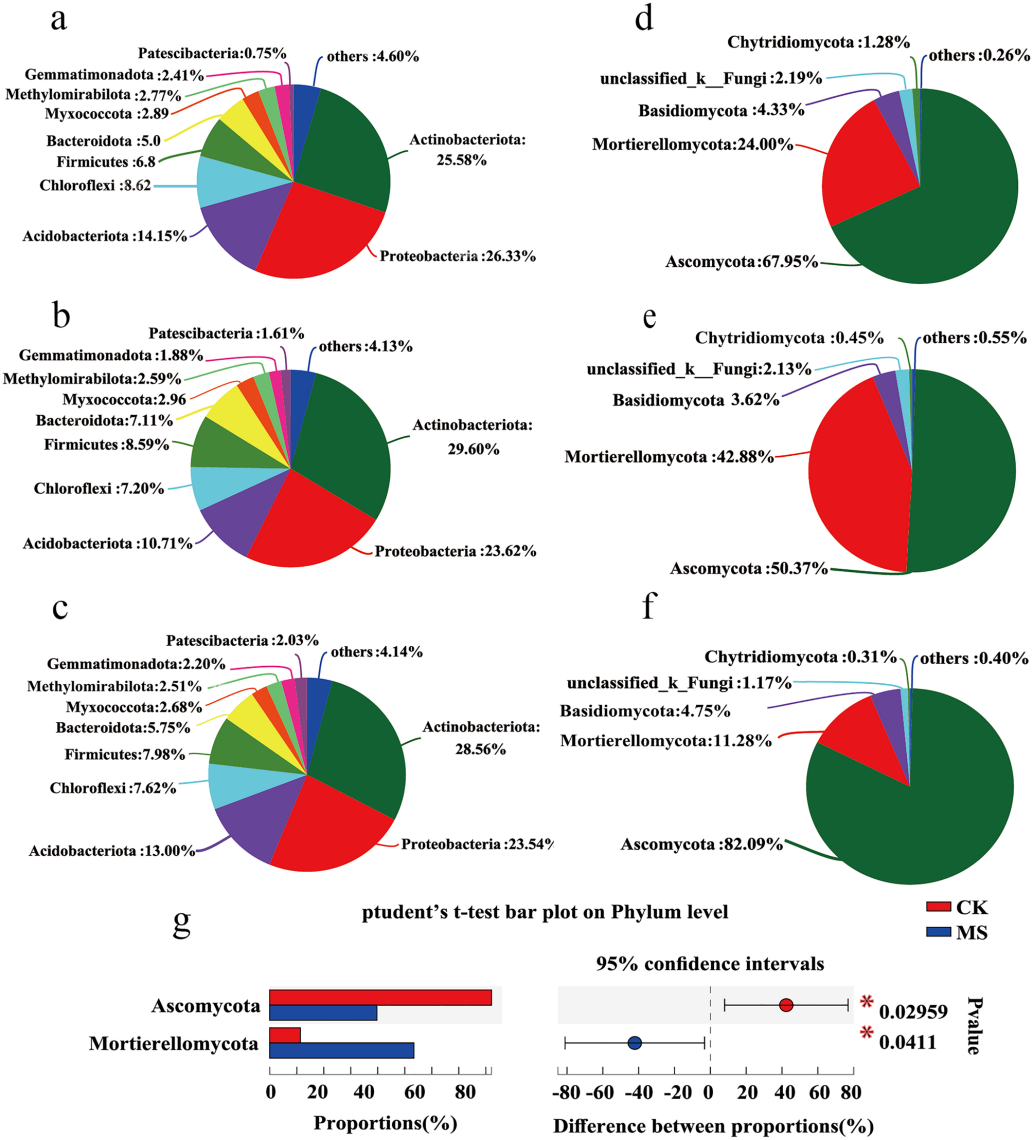
Differences in community structure between groups. Relative abundance (%) of bacterial (**a**–**c**) and fungus (**d**–**f**) community composition at the phylum level among different treatments: (**a**,**d**) CK, (**b**,**e**) SE, (**c**,**f**) MS. (**g**) The significant differences in bacterial and fungal groups at the phylum level among the treatments (Student’s *t*-test bar plot on phylum level). The rightmost is p value, *0.01 < p ≤ 0.05.

The analysis of relative abundance differences at the phylum level revealed only small differences in the bacterial communities in wheat rhizosphere between the treatments (Fig. 6a–c), with nine phyla detected. In the CK, SE and MS groups, Actinobacteriota (25.58%, 29.60%, 28.56%), Proteobacteria (26.33%, 23,62%, 23.54%), and Acidobacteriota (14.15%, 10.71%, 13.00%) were the dominant bacterial phyla, followed by Firmicutes (6.8%, 8.59%, 7.98%)), Chlorobacteria (8.62%, 7.20%, 7.62%), Bacteroidetes (5.0%, 7.11%, 5.75%), Myxococcus (2.89%, 2.96%, 2.68%), Methylomirabilota (2.77%, 2.59%, 2.51%), Gemmatimonadota (2.41%, 1.88%, 2.20%) and Patescibacteria (0.75%, 1.61%, 2.03%). The analysis revealed that although the SE and MS treatments did not significantly affect the abundance and diversity of bacterial community, they significantly affected the abundance and diversity of wheat rhizosphere fungal communities (Fig. 6d–f). As shown in Fig. 6d,f, the relative abundance of Ascomycota and Mortierellomycota was significantly different between MS and CK samples. The relative abundance of Ascomycota decreased from 82.09% in the CK group to 50.37% in the MS group. The relative abundance of Mortierellomycota significantly increased from 11.28% in the CK group to 42.88% in the MS group (*p* < 0.05, Fig. 6g).

**Figure 7:**
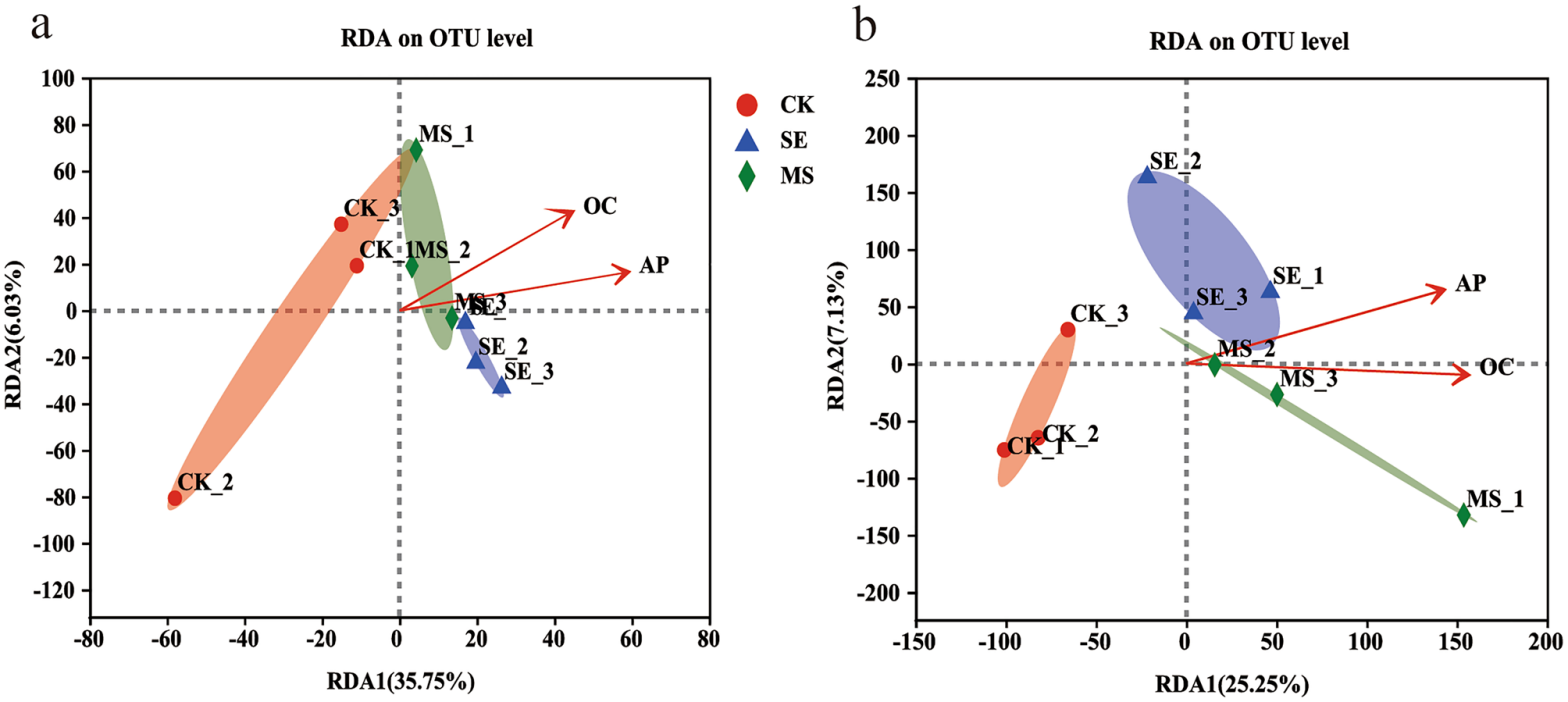
Redundancy analysis of (**a**) bacteria and (**b**) fungus at OTU level in different treatments.

The results of the redundancy analysis of soil bacterial community composition and environmental factors are shown in Fig. 7. The main physicochemical factors affecting soil bacterial community composition were effective phosphorus and sucrase (*p* < 0.05), with the former having the most significant effect on the bacterial community structure (r^2^ = 0.0.6876*, p* = 0.028, Table S1). The redundancy analysis of rhizosphere soil fungal community composition and environmental factors revealed that the following physicochemical factors affected the soil fungal community structure: effective phosphorus, organic matter, effective potassium, pH, urease, phosphatase, and sucrase (*p* < 0.05); of these factors, sucrase had the most significant effect on the fungal community structure (r^2^ = 0.8494, *p* = 0.005, Table S1).

## Discussion

Kelp is a cheap and abundant resource in coastal agricultural areas. A large amount of KSW is generated during the kelp processing. The direct discharge of untreated wastewater will lead to waste of resources, affect aquatic resources and aquaculture, cause harm to the Marine environment, worsen the quality of seawater, and even endanger human health. Therefore, how to make reasonable use of KSW has both economic and environmental benefits. Here, we applied KSW at appropriate concentrations to wheat seedlings and verified the growth-promoting effects of KSW and the mixture of *B. methylotrophicus* M4-1 and KSW on the growth of wheat seedlings as well as the structure and diversity of the microbial community in wheat seedlings rhizosphere. We confirmed that the mixture of KSW and *B. methylotrophicus* M4-1 improved the soil physicochemical properties, promoted wheat growth, and significantly increased the dry and fresh weights and height of the wheat seedlings. Moreover, the combination improved the rhizosphere microbial community structure, which further promoted the growth of wheat seedlings. These findings suggest that the combination could be used as a natural fertilizer for wheat, providing an environmentally friendly approach to treat kelp wastewater.

The application of rhizobacteria, specifically PGPR, as an alternative to chemical pesticides has recently gained significant attention in the scientific community. Upon application of microbial fertilizers to the seed, plant, rhizosphere, and the soil, PGPR usually colonize the rhizosphere or plant interior and increase the supply of nutrients to the host plant, thus promoting plant growth^39^. PGPR affect plant growth by improving the physicochemical properties of the soil as well as by producing a variety of metabolites, such as phytohormones (growth hormone, cytokinin, gibberellin, and ethylene), antibiotics, siderophores, extracellular polysaccharides, and ACC deaminase^40,41^. These compounds stimulate the increase in plant root length, root surface area, and root tip number, thereby increasing nutrient uptake by the plant^42–44^. Of note, KSW in this study are rich in phytohormones, such as indoleacetic acid and abscisic acid.

In addition to PGPR, biostimulants can also promote plant growth and suppress plant soil-borne diseases^12,45^. The term biostimulant was first introduced in 2007 by Kauffman et al., who considered biostimulants as substances other than fertilizers that promote plant growth when applied in small amounts^46^. The KSW used in the current study contains a variety of biostimulants. Specifically, it is rich in plant growth regulators (phytohormones), minerals, and trace elements, quaternary ammonium molecules (e.g., betaines), polyuronides (e.g., alginates or fucoidans), and lipid-based molecules (e.g., sterols). Biostimulants enhance root formation and elongation, increase nutrient uptake, improve seed germination and crop establishment, increase cation exchange, reduce leaching, detoxify heavy metals, and stimulate the plant immune system in response to stressors^47^. In addition to promoting plant growth, biostimulants can suppress soil-borne diseases in plants^12,45^.

Hernandez et al. confirmed that, the number of petioles and root length of strawberry significantly increased after treatment with *B. methylotrophicus*^48^. A similar result was reported by Yao et al.^11^, who showed that betaine and phytohormones shortened the ripening time and increased the yield of tomato by increasing the leaf area, increasing the intensity of photosynthesis, and promoting gas exchange in the leaf. In the current study, the mixture of KSW and strain M4-1 significantly increased the dry weight and fresh weight of wheat seedlings (*p* < 0.05, Fig. 1), presumably because the phytohormones and other substances produced by strain M4-1 promoted the number of root tips, root surface area, and root length of wheat seedlings, thus improving the uptake and utilization of various soil nutrients and, hence, the growth of wheat seedlings.

The physical and chemical properties of the soil are critical to plant growth. The pH, water, nitrogen, phosphorus, and potassium content; and activity of various enzymes directly affect plant growth. In the current study, the addition of KSW and the combination of KSW with strain M4-1 resulted in significant changes in all soil indicators relative to CK conditions. PGPR play an important role in soil nutrient transformation, promoting the transformation of complex organic molecules, facilitating the acquisition of environmental resources by the plant, and enhancing the acquisition and assimilation of nutrients by plant^49^. In the current study, the available potassium content in MS samples was significantly higher than that in CK samples (*p* < 0.05, Fig. 2c). This result can be explained by the fact that organic acids produced by PGPR interact with phosphorus and potassium compounds present in the soil, which are otherwise not easily accessible by the plant, and convert these elements into available phosphorus and available potassium, respectively, thereby improving the uptake and utilization of phosphorus and potassium by the plant^50,51^. A small increased in soil pH greatly alters the solubility of silicate and changes the amount of available potassium in the soil^52^. Consistent with these data, available potassium levels in MS samples were significantly higher than those in CK samples (*p* < 0.05). Additionally, strain M4-1 produces organic acids that release K from mineral complexes by forming chelates with Fe^2+^, Al^3+^, Si^4+^, and Ca^2+^ ions bound to K minerals^53^.

In addition to increasing the content of nutrients in the soil, PGPR and KSW improved the activity of various soil enzymes. We showed that the addition of KSW alone or together with strain M4-1 altered the activities of various soil enzymes (sucrase, urease, phosphatase, and catalase), with a significant increase in urease activity in MS samples compared to the CK group. Urease, a key enzyme that regulates soil nitrogen transformation, is mainly produced by plants and microbes and plays a key role in nutrient cycling^54^. Soil enzymes are among the most active organic components of soil aggregates^55^. They are used for the assessment of soil fertility and the rate of chemical composition change^17^. Furthermore, soil enzyme activity is often used as an indicator of microbial growth in the soil. The observed changes in the various enzyme activities in MS samples may be associated with changes in soil physical properties caused by the application of KSW and PGPR, which increased soil fertility and created an environment conducive to microbial growth, thus affecting soil enzyme activities^56^.

High-throughput sequencing is widely used in biological applications, such as the assessment of soil microbial diversity, which is a key factor affecting soil health and quality. In the current study, total DNA was isolated from wheat seedlings rhizosphere soil from different treatment groups, and the 16S rRNA and ITS regions were sequenced. The analysis revealed that the mixture of KSW and PGPR significantly affected (*p* < 0.05) fungal abundance and diversity, while the mixture of KSW significantly affected (*p* < 0.05) fungal abundance. The KSW alone or the mixture of KSW and PGPR affected the abundance and diversity of rhizosphere bacteria, however, the effect was not significant.

At the bacterial phylum level, Actinobacteria, Proteobacteria, and Acidobacteria were dominant, followed by Firmicutes, Chloroflexi, Bacteroidetes, Myxococcota, and Mehylomirabilota, Gemmatimonadota, and Patescibacteria. Actinomycetes are the main taxa of soil microorganisms that participate in the recycling of organic matter in the environment by secreting hydrolytic enzymes and also produce metabolites that promote plant growth, such as iron carriers and growth factors^57,58^. The relative abundance of the Actinomycetes in the rhizosphere soil of wheat in the treatment group of this experiment was higher than that of the control group, indicating that KSW and M4-1 can effectively increase the nutrient level of the soil and promote plant growth. In rhizosphere soils, the Proteobacteria is often associated with the nutrient content of the soil and is involved in nitrogen fixation, organic matter decomposition and promotion of plant growth^59^. Acidobacteria tend to be associated with the nutrient content of the soil^27,60^. Acidobacteria plays an important role in soil carbon and nitrogen cycling^61^ and greatly affects plant growth traits and yield. For instance, Tao et al.^62^ reported that Acidobacteria and Verrucomicrobia are significantly positively correlated with grain yield of maize fertilized with different green manures (*p* < 0.01). Bacteroidetes produces large amounts of extracellular polysaccharides, which protect the crop root system. In addition, extracellular polysaccharides improve the rhizosphere microenvironment and promote the uptake of trace elements by the plant^63,64^. The relative abundance of Bacteroidetes in wheat rhizosphere soil in the MS group was higher than that in the CK group (Fig. 6a–c), indicating that strain M4-1 increased the nutrient content of the rhizosphere soil.

At the fungal phylum level, five phyla were detected, namely, Ascomycota, Mortierellomycota, Basidiomycota, Chytridiomycota, and Unclassified Fungi (Fig. 6d–f). Compared with CK samples, the relative abundance of Ascomycota and Mortierellomycota was significantly different in MS samples (*p* < 0.05, Fig. 6). Ascomycota is a phylum with the largest fungal variety, with most diverse reproductive forms and morphology, encompassing over 64,000 species^65,66^. However, some Ascomycetes can infect and damage plants^67^, including crops, leading to serious economic and health problems globally^68,69^. In the current study, the relative abundance of Ascomycota in the rhizosphere soil of wheat in MS and SE samples was notably lower than that in CK samples (Fig. 6d–f; 50.37% in MS samples, 67.95% in SE samples, and 82.09% in CK samples). The relative abundance of Ascomycota in MS samples was only 31.72% of that in CK samples. This result indicates that the addition of KSW together with strain M4-1 significantly reduced the relative abundance of the plant pathogenic fungi Ascomycota in the soil (*p* < 0.05, Fig. 6g). In addition to Ascomycota, the relative abundance of Mortierellomycota also differed significantly among the groups (*p* < 0.05, Fig. 6g). The relative abundance of Mortierellomycota was 42.88% in MS samples, 24.00% in SE samples, and 11.28% in CK samples. *Mortierella* is a common environmental filamentous fungus species^70^ found in different soils, the rhizosphere, rivers, and lakes^71,72^. Mortierellomycota decomposes a variety of complex organic compounds and inhibits the growth and reproduction of some fungal and bacterial pathogens^73^. Furthermore, Mortierellomycota synthesizes gibberellin, indoleacetic acid, and ACC deaminase, which promote the growth of tomato and wheat^72,74^.

Figures 5 and 6 show that the fungal community structure of the MS samples differed from that of the SE samples due to the addition of *B. methylotrophic* M4-1 suspension to the MS treatment. Studies have shown that, *B. methylotrophicus* secrete several antifungal metabolites to inhibit the growth of pathogens^75^, such as inhibition of suppression of maize stalk rot^76^, tomato bacterial wilt^77^ and other pathogens. *B. methylotrophicus* M4-1 in this study was able to secrete siderophores (37.21 mg l^−1^), protease (670.82 U mL^−1^) and cellulase (36.27 U mL^−1^)^78^. Cellulases and proteases can hydrolyze fungal cell walls and inhibit the growth of fungal pathogens^79^. Siderophores play a significant role in the biological control mechanism against certain phytopathogens. Siderophores bind with the iron tightly and create Fe competition in the rhizospheric zone, which reduce the bioavailable iron for the plant pathogens and decreases pathogenic microbe abundance^80,81^. In summary, the M4-1 suspension added to the MS treatment was able to disturb the microbial community structure composition of the inter-rhizosphere soil, especially that of fungal microorganisms.

## Conclusion

The present study aimed to verify the promotion effect of KSW and strain M4-1 mixture on the growth of wheat seedlings and the effect on the rhizosphere microbial community structure of wheat seedlings. The mixture of KSW and strain M4-1 improves the physicochemical properties of the soil by increasing the content of available phosphorus, available nitrogen, available potassium, and organic matter; altering the soil pH; and enhancing soil enzyme activity compared to the control. The mixture of KSW and strain M4-1 promotes the growth of wheat seedlings and significantly increases the dry and fresh weight of wheat seedlings while improving microbial abundance and diversity in wheat seedlings rhizosphere soil, reducing the relative abundance of pathogenic fungi, and increasing the relative abundance of beneficial bacteria, further promoting the growth of wheat seedlings and improving nutrient utilization efficiency of wheat seedlings. Here, we suggest using a mixture of KSW and strain M4-1 as biostimulants for wheat seedlings, which would solve the problem of difficult treatment of KSW in production, conserve agricultural water, and also alleviate the environmental pollution caused by direct discharge of KSW.

## Materials and methods

### Ethics

The collection of plants material complies with relevant institutional, national and international guidelines and legislation. The samples in the study were collected on private land where the owner allowed the study. The experimental materials did not involve any humans or animals. All methods were performed in accordance with the relevant guidelines and regulations.

### Preparation and composition analysis of KSW

The KSW was provided by Qingdao Mingyue Company (Qingdao, Shandong). The KSW was obtained after 20 h of kelp soaking (kelp to water ratio 1:20) used in the production of alginate, algal functional sugar alcohols, algal functional food ingredients, etc. The components of the original solution of KSW were analyzed by Qingdao Feiyoute Testing Company (Qingdao, Shandong), and the findings are shown in Table 1.

**Table 1:** Composition analysis table of KSW.

The test items	Results	Detection limit	Loq	position	Test method/basis
Fucoidin	531	25	50	mg/kg	FUT-SOP(Y)-52.1
Mannitol	1.00	0.025	0.05	%	FUT-SOP(Y)-43.1
Crude protein	0.05	–	–	g/100 g	GB 5009.5-2016
Betaine	5.14 × 10^3^	10	20	µg/kg	FUT-SOP(Y)-34.1
The total polyphenol	8.43	–	–	mg/kg	FUT-SOP(L)-15.1
Indole acetic acid	9.1	0.3	1.0	µg/kg	FUT-SOP(Y)-34.1
Abscisic acid	1.3	0.3	1.0	µg/kg	FUT-SOP(Y)-34.1
N	202	–	0.05	mg/L	HJ 636-2012
p	173	–	0.01	mg/L	GB/T 11893-1989
K	4.50 × 10^3^	–	0.05	mg/L	FUT-SOP(W)-1.1
I	27.1	–	0.1	mg/L	GB/T 13882–2010
Ca	80.9	–	0.02	mg/L	FUT-SOP(W)-1.1
Mg	149	–	0.003	mg/L	FUT-SOP(W)-1.1
Fe	2.22	–	0.02	mg/L	FUT-SOP(W)-1.1
Zn	0.120	–	0.004	mg/L	FUT-SOP(W)-1.1
Mn	0.315	–	0.004	mg/L	FUT-SOP(W)-1.1
Glutamate	0.010	–	0.01	g/100 g	GB 5009.124-2016

–, N/A.

### Microbial inoculum preparation

*Bacillus methylotrophicus* M4-1 was isolated from the inter-rhizosphere soil of wheat in the saline zone of the Yellow River Delta, Shandong Province, China (118°49ʹ15″E, 37°24′31″N). It is a methylotrophic *Bacillus* strain, a gram-positive, facultatively aerobic bacterium. The detailed physiological and biochemical characteristics of this strain have been described by Ji et al.^78^. The NCBI accession number of M4-1 is MN938176.1.

Strain M4-1 was used to inoculate Luria–Bertani LB liquid medium and cultured in a shaking incubator at 30 °C with 200 rpm for 12 h to obtain the seed solution. The seed solution was inoculated into a laboratory-optimized fermentation medium (per liter of water: glucose, 20.0 g; peptone, 36.0 g; MgSO_4_, 3.2 g; K_2_HPO_4_, 5.6 g; KH_2_PO_4_, 2.8 g), at 2% (v/v) inoculum size, and incubated at 30 °C with 200 rpm shaking until the spore yield rate exceeded 90%. The spore yield rate was evaluated under an optical microscope (NIKON, Tokyo, JPN) with crystal violet staining^82^. The microbial pellet cells in the fermentation broth was collected and centrifuged at 4000 rpm for 30 min. The microbial pellet was resuspended in sterile deionized water for cell density of 1 × 10^9^ cfu/mL^83^.

### Pot experiment

Wheat cv jimai 21 seeds (provided by the College of Agriculture, Shandong Agricultural University) were washed with water, soaked in 75% ethanol for 10 min, then soaked in 30% sodium hypochlorite for 30–60 s, and finally washed 5–6 times with sterile water and dried. Uniform wheat seeds were selected and sown into pots (17 cm inner diameter), 12 seeds per pot. The soil used for the pot experiment were from an experimental field of Shandong Agricultural University (117°16ʹE, 36°17ʹN). After the wheat seedlings were approximately 10 cm long, the plants were watered with five different dilutions of KSW (20 mL) or the mixture of kelp-soaked wastewater at different dilutions and M4-1 bacterial suspension (1 × 10^9^ cfu/mL, 10 mL), while the control group (CK) was only irrigated using 30 mL water. After that all groups were irrigated every 7 days with 30 mL water. Randomized complete block design was used in this study.

Kelp was soaked for 20 h according to the kelp to water ratio of 1:20 to obtain KSW stock solution. Measure 5 portions of KSW stock solution 0.1 L, add water 0.9 L, 1.9 L, 3.9 L, 7.9 L, 11.9 L respectively, and mix thoroughly to obtain the KSW with the dilution multiple of 10, 20, 40, 80 and 120 in order. The pH values of KSW with different dilutions (10, 20, 40, 80, 120) were 6.19, 6.46, 6.74, 6.97, 7.04, respectively. Six replicates were set up for each group. Potted were grouped as in Table 2.

**Table 2:** Group design of pot experiment and additives in each group.

Groups	Dilution multiple of KSW	Volume of KSW (mL)	Volume of water (mL)	Volume of strain M4-1 suspension (mL)
SA	10	20	10	–
MP			–	10
SC	20	20	10	
MQ			–	10
SD	40	20	10	–
MR			–	10
SE	80	20	10	–
MS			–	10
SF	120	20	10	–
MT			–	10
CK	–	–	30	–

*KSW* kelp-soaking wastewater, – N/A.

### Collection of wheat rhizosphere soil

After 35 days of treatment, the wheat seedlings were uprooted, and the roots were gently shaken to remove large soil particles. The soil tightly attached to the root surface was the rhizosphere soil^84^. The rhizosphere soil from two pots of wheat seedlings combined, for three repeat samples. The rhizosphere soil was passed through a 2-mm sieve. A portion of the soil sample was air-dried for the determination of physical and chemical properties, and another portion was stored at − 80 °C for the extraction of soil DNA. Three replicates were prepared for each treatment. Wheat seedlings were collected for growth and biomass surveys, and each pot was used as a replicate with 6 replicates in each group.

### Extraction and PCR amplification of total soil microbial DNA

Total soil microbial DNA was extracted using FastDNA SPIN Kit for Soil. Purity of soil DNA was confirmed using 1% agarose gel electrophoresis. Quality-controlled soil DNA samples were sequenced by high-throughput sequencing. Three replicates were prepared for each treatment.

Universal primers specific to the bacterial 16S rDNA V3–V4 region (338F, 5′-barcode-ACTCCTACGGGAGGCAGCA-3′ and 806R, 5′-GGACTACHVGGGTWTCTAAT-3′) and to the fungal rDNA-ITS sequence (5′-barcode-CTTGGTCATTTAGAGGAAGTAA-3′ and 2043R, 5′-GCTGCGTTCTTCATCGATGC-3′) were used for PCR amplification. PCR was set up as follows: 4 μL of 5 × FastPfu buffer, 2 μL of 2.5 mM dNTPs, 0.8 μL of positive primer (5 μM), 0.8 μL of negative primer (5 μM), 0.4 μL of fast-PFU polymerase, 10 ng template DNA 1, and ddH_2_O for a final volume of 20 μL. The reaction parameters for the amplification of the bacterial V3–V4 region were as follows: pre-denaturation at 95 °C for 2 min; 40 cycles of denaturation at 95 °C for 30 s, annealing at 55 °C for 30 s, and extension at 72 °C for 30 s; and final extension at 72 °C for 5 min. For the amplification of the fungal rDNA-ITS sequence, PCR was performed as follows: pre-denaturation at 95 °C for 3 min; 40 cycles of denaturation at 95 °C for 30 s, annealing at 55 °C for 30 s, and extension at 72 °C for 45 s; and final extension at 72 °C for 10 min. Sample quality was examined by 2% agarose gel electrophoresis. The DNA bands were extracted and PCR products were recovered using an AxyPrep DNA Gel Extraction Kit (Axygen Biosciences, Union City, CA, USA). Finally, the PCR products were quantified using the QuantiFluor™-ST Blue Fluorescence Quantification System (Promega). Equimolar amounts of the purified amplicons were pooled and subjected to paired-end sequencing using an Illumina MiSeq instrument according to standard protocols.

### Processing of sequencing data

Double-ended sequence data were obtained by MiSeq sequencing. First, pairs of reads were combined based on overlaps between the reads. QIIME (version 1.9.1) (http://qiime.sourceforge.net/) was used for quality read filtering. After the sample data were collated, OTU clustering at 97% similarity level and species taxonomic analyses were performed using UPARSE (version 7.1). Following OTU clustering, sample diversity indices were calculated using Mothur (version 1.30.2) software to compare the relative microbial diversity in samples. The 16S RNA read data were compared with information in the Silva (SSU123) 16S rRNA database, and RDP classifier was used to analyze 16S RNA reads in bacterial groups at different classification levels. The RDP classifier was also used to classify the ITS rDNA gene reads into fungal groups at different classification levels by comparing with UNITE 7.0/ITS database, at a confidence threshold of 0.7. UPGMA clustering analysis and PCoA were used to analyze the similarity between samples. Perl scripts were used to generate Venn diagrams to compare species composition of samples.

### Determination of wheat growth, physical and chemical properties of the soil

The height and fresh weight of wheat seedlings in the treatment and control groups were determined. Thereafter, all wheat seedlings plants were baked in an oven at 80 °C until a constant weight was achieved as dry weight.

The available phosphorus (AP) was assayed by extraction with sodium bicarbonate^85^, Available potassium (AK) was assayed by the flame photometry method^86^, organic matter (OM) was assayed by the chromic acid titration method^87^, and alkali-hydrolyzed nitrogen was assayed by the alkali diffusion method. Soil pH was measured using a pH meter (METTLER TOLEDO, Shanghai, CHN). Activities of sucrase was determined by a colorimetric method using 3,5-dinitrosalicylic acid^88^. Urease was determined by sodium phenol-sodium hypochlorite colorimetric method^88,89^. Phosphatase was determined by benzene disodium phosphate colorimetric method^90^. Catalase was determined by potassium permanganate titration method^88,91^.

### Statistical analysis

Data were expressed as the mean ± standard deviation (SD). Statistical analysis of plant and soil parameters was performed using ANOVA. In the process of one-way ANOVA, if the data do not meet the normal distribution, rank sum test is used. If variance is not uniform, Welch test is used. SPSS26.0 software (SPSS 26.0, SPSS, Chicago, IL, USA) was used for data statistics, differences in mean values were considered significant when *p* < 0.05.

### Supplementary Information

Supplementary Information.

## Data Availability

The datasets presented in this study can be found in online repositories. The names of the repository/repositories and accession number(s) can be found at: NCBI—PRJNA839880, PRJNA839884.

## References

[1] Wood CG, Clair G (1974). Seaweed extracts: A unique ocean resource. J. Chem. Educ..

[2] Poblete-Castro I, Hoffmann S-L, Becker J, Wittmann C (2020). Cascaded valorization of seaweed using microbial cell factories. Curr. Opin. Biotechnol..

[3] Tilman D (2001). Forecasting agriculturally driven global environmental change. Science.

[4] Zhang Y, Wang J, Dai C (2021). The adjustment of China's grain planting structure reduced the consumption of cropland and water resources. Int. J. Environ. Res. Public Health.

[5] Sun Y, Hu R, Zhang C (2019). Does the adoption of complex fertilizers contribute to fertilizer overuse? Evidence from rice production in China. J. Clean. Prod..

[6] Garai S, Brahmachari K, Sarkar S, Kundu R, Pramanick B (2019). Crop growth and productivity of rainy maize-garden pea copping sequence as influenced by *Kappaphycus* and *Gracilaria* saps at alluvial soil of West Bengal, India. Curr. J. Appl. Sci. Technol..

[7] Ashour M (2021). Impact of commercial seaweed liquid extract (TAM(^®^)) biostimulant and its bioactive molecules on growth and antioxidant activities of hot pepper (*Capsicum*
*annuum*). Plants (Basel).

[8] Sangha JS (2014). Seaweeds (macroalgae) and their extracts as contributors of plant productivity and quality. Adv. Bot. Res..

[9] Battacharyya D, Babgohari MZ, Rathor P, Prithiviraj B (2015). Seaweed extracts as biostimulants in horticulture. Sci. Hortic..

[10] Biris-Dorhoi E-S (2020). Macroalgae—A sustainable source of chemical compounds with biological activities. Nutrients.

[11] Yao Y, Wang X, Chen B, Zhang M, Ma J (2020). Seaweed extract improved yields, leaf photosynthesis, ripening time, and net returns of tomato (*Solanum*
*lycopersicum* Mill.). ACS Omega.

[12] Rengasamy KRR, Kulkarni MG, Pendota SC, Van Staden J (2016). Enhancing growth, phytochemical constituents and aphid resistance capacity in cabbage with foliar application of eckol—A biologically active phenolic molecule from brown seaweed. New Biotechnol..

[13] Elansary HO (2016). Enhancement of *Calibrachoa* growth, secondary metabolites and bioactivity using seaweed extracts. BMC Complement Altern. Med.

[14] Garde-Cerdán T (2021). Influence of seaweed foliar application to *Tempranillo* grapevines on grape and wine phenolic compounds over two vintages. Food Chem..

[15] Cook J, Zhang J, Norrie J, Blal B, Cheng Z (2018). Seaweed extract (Stella Maris^®^) activates innate immune responses in *Arabidopsis*
*thaliana* and protects host against bacterial pathogens. Mar. Drugs.

[16] Trivedi K (2021). Structural and functional changes in soil bacterial communities by drifting spray application of a commercial red seaweed extract as revealed by metagenomics. Arch. Microbiol..

[17] Chen Y (2020). Impact of short-term application of seaweed fertilizer on bacterial diversity and community structure, soil nitrogen contents, and plant growth in maize rhizosphere soil. Folia Microbiol..

[18] du Jardin P (2015). Plant biostimulants: Definition, concept, main categories and regulation. Sci. Hortic..

[19] Vacheron J (2013). Plant growth-promoting rhizobacteria and root system functioning. Front. Plant Sci..

[20] Vessey JK (2003). Plant growth promoting rhizobacteria as biofertilizers. Plant Soil.

[21] Gouda S (2018). Revitalization of plant growth promoting rhizobacteria for sustainable development in agriculture. Microbiol. Res..

[22] Vejan P, Abdullah R, Khadiran T, Ismail S, Nasrulhaq Boyce A (2016). Role of plant growth promoting rhizobacteria in agricultural sustainability—A review. Molecules.

[23] Backer R (2018). Plant growth-promoting rhizobacteria: Context, mechanisms of action, and roadmap to commercialization of biostimulants for sustainable agriculture. Front. Plant Sci..

[24] Sayyed RZ, Reddy MS, Antonius S (2019). Plant Growth Promoting Rhizobacteria (PGPR): Prospects for Sustainable Agriculture Prospects for Sustainable Agriculture.

[25] He A (2021). Two PGPR strains from the rhizosphere of *Haloxylon*
*ammodendron* promoted growth and enhanced drought tolerance of ryegrass. Plant Physiol. Biochem..

[26] Nawaz A (2020). Potential of salt tolerant PGPR in growth and yield augmentation of wheat (*Triticum*
*aestivum* L.) under saline conditions. Front. Microbiol..

[27] Ji C (2021). Effect of *Bacillus velezensis* JC-K3 on endophytic bacterial and fungal diversity in wheat under salt stress. Front. Microbiol..

[28] Ali S, Khan N (2021). Delineation of mechanistic approaches employed by plant growth promoting microorganisms for improving drought stress tolerance in plants. Microbiol. Res..

[29] Guo J (2020). Prospects and applications of plant growth promoting rhizobacteria to mitigate soil metal contamination: A review. Chemosphere.

[30] Fernando WGD, Nakkeeran S, Zhang Y (2005). Biosynthesis of Antibiotics by PGPR and Its Relation in Biocontrol of Plant Diseases.

[31] Lugtenberg B, Chin-A-Woeng T, Bloemberg GV (2002). Microbe–plant interactions: Principles and mechanisms. Antonie van Leeuwenhoek.

[32] Somers E, Vanderleyden J, Srinivasan M (2008). Rhizosphere bacterial signalling: A love parade beneath our feet. Crit. Rev. Microbiol..

[33] Syed Nabi RB (2021). Evaluation potential of PGPR to protect tomato against Fusarium wilt and promote plant growth. PeerJ.

[34] Rose S, Parker M, Punja ZK (2003). Efficacy of biological and chemical treatments for control of fusarium root and stem rot on greenhouse cucumber. Plant Dis..

[35] Paungfoo-Lonhienne C, Redding M, Pratt C, Wang W (2019). Plant growth promoting rhizobacteria increase the efficiency of fertilisers while reducing nitrogen loss. J. Environ. Manag..

[36] Steen AD (2019). High proportions of bacteria and archaea across most biomes remain uncultured. ISME J..

[37] Alteio LV (2021). A critical perspective on interpreting amplicon sequencing data in soil ecological research. Soil Biol. Biochem..

[38] Caporaso JG (2012). Ultra-high-throughput microbial community analysis on the Illumina HiSeq and MiSeq platforms. ISME J..

[39] Mahanty T (2017). Biofertilizers: A potential approach for sustainable agriculture development. Environ. Sci. Pollut. Res..

[40] Grover M, Ali SZ, Sandhya V, Rasul A, Venkateswarlu B (2011). Role of microorganisms in adaptation of agriculture crops to abiotic stresses. World J. Microbiol. Biotechnol..

[41] Khan N, Mehmood A, Ali S, Shahid MA, Rakshit A (2021). Biofertilizers.

[42] Gamez R (2019). Screening, plant growth promotion and root colonization pattern of two rhizobacteria (*Pseudomonas*
*fluorescens* Ps006 and *Bacillus*
*amyloliquefaciens* Bs006) on banana cv. Williams (*Musa*
*acuminata* Colla). Microbiol. Res..

[43] Sureshbabu K, Amaresan N, Kumar K (2016). Amazing multiple function properties of plant growth promoting rhizobacteria in the rhizosphere soil. Int. J. Curr. Microbiol. Appl. Sci..

[44] Egamberdieva D, Kucharova Z (2009). Selection for root colonising bacteria stimulating wheat growth in saline soils. Biol. Fertil. Soils.

[45] Kapoore RV, Wood EE, Llewellyn CA (2021). Algae biostimulants: A critical look at microalgal biostimulants for sustainable agricultural practices. Biotechnol. Adv..

[46] Kauffman GL, Kneivel DP, Watschke TL (2007). Effects of a biostimulant on the heat tolerance associated with photosynthetic capacity, membrane thermostability, and polyphenol production of perennial ryegrass. Crop Sci..

[47] Shahrajabian MH, Chaski C, Polyzos N, Petropoulos SA (2021). Biostimulants application: A low input cropping management tool for sustainable farming of vegetables. Biomolecules.

[48] Vicente-Hernández A (2018). Bacillus methylotrophicus M4–96 stimulates the growth of strawberry (*Fragaria*×*ananassa* 'Aromas') plants in vitro and slows *Botrytis*
*cinerea* infection by two different methods of interaction. J. Plant Growth Regul..

[49] Santoyo G, Moreno-Hagelsieb G, del Carmen Orozco-Mosqueda M, Glick BR (2016). Plant growth-promoting bacterial endophytes. Microbiol. Res..

[50] Cardoso AF (2021). *Bacillus cereus* improves performance of Brazilian green dwarf coconut palms seedlings with reduced chemical fertilization. Front. Plant Sci..

[51] Qu Q (2020). Rhizosphere microbiome assembly and its impact on plant growth. J. Agric. Food Chem..

[52] Sheng XF (2005). Growth promotion and increased potassium uptake of cotton and rape by a potassium releasing strain of *Bacillus edaphicus*. Soil Biol. Biochem..

[53] Meena VS, Maurya BR, Verma JP (2014). Does a rhizospheric microorganism enhance K^+^ availability in agricultural soils?. Microbiol. Res..

[54] Wang H, Wu J, Li G, Yan L (2020). Changes in soil carbon fractions and enzyme activities under different vegetation types of the northern Loess Plateau. Ecol. Evol..

[55] Marx MC, Wood M, Jarvis SC (2001). A microplateuorimetric assay for the study of enzyme diversity in soils. Soil Biol. Biochem..

[56] Burns RG (2013). Soil enzymes in a changing environment: Current knowledge and future directions. Soil Biol. Biochem..

[57] Bhatti AA, Haq S, Bhat RA (2017). Actinomycetes benefaction role in soil and plant health. Microb. Pathog..

[58] Bao Y (2021). Important ecophysiological roles of non-dominant Actinobacteria in plant residue decomposition, especially in less fertile soils. Microbiome.

[59] Kim H-S, Lee S-H, Jo HY, Finneran KT, Kwon MJ (2021). Diversity and composition of soil *Acidobacteria* and *Proteobacteria* communities as a bacterial indicator of past land-use change from forest to farmland. Sci. Total Environ..

[60] Beckers B, Beeck M, Weyens N, Boerjan W, Vangronsveld J (2017). Structural variability and niche differentiation in the rhizosphere and endosphere bacterial microbiome of field-grown poplar trees. Microbiome.

[61] Jiménez DJ (2012). Structural and functional insights from the metagenome of an acidic hot spring microbial planktonic community in the Colombian Andes. PLoS One.

[62] Tao J (2017). Maize growth responses to soil microbes and soil properties after fertilization with different green manures. Appl. Microbiol. Biotechnol..

[63] Chen L (2021). Effects of growth-promoting rhizobacteria on maize growth and rhizosphere microbial community under conservation tillage in Northeast China. Microb. Biotechnol..

[64] Madhaiyan M (2010). *Mucilaginibacter*
*gossypii* sp. nov. and *Mucilaginibacter*
*gossypiicola* sp. nov., plant-growth-promoting bacteria isolated from cotton rhizosphere soils. Int. J. Syst. Evol. Microbiol..

[65] Blackwell M (2011). The fungi: 1, 2, 3 … 5.1 million species?. Am. J. Bot..

[66] Ma A (2013). Ascomycota members dominate fungal communities during straw residue decomposition in arable soil. PLoS One.

[67] Zeilinger S (2016). Friends or foes? Emerging insights from fungal interactions with plants. FEMS Microbiol. Rev..

[68] Oide S (2006). NPS6, encoding a nonribosomal peptide synthetase involved in siderophore-mediated iron metabolism, is a conserved virulence determinant of plant pathogenic ascomycetes. Plant Cell.

[69] Alshannaq A, Yu J-H (2017). Occurrence, toxicity, and analysis of major mycotoxins in food. Int. J. Environ. Res. Public Health.

[70] Nagy LG (2011). Where is the unseen fungal diversity hidden? A study of *Mortierella* reveals a large contribution of reference collections to the identification of fungal environmental sequences. New Phytol..

[71] Gonçalves VN, Vaz ABM, Rosa CA, Rosa LH (2012). Diversity and distribution of fungal communities in lakes of Antarctica. FEMS Microbiol. Ecol..

[72] Ozimek E (2018). Synthesis of indoleacetic acid, gibberellic acid and ACC-deaminase by strains promote winter wheat seedlings growth under different conditions. Int. J. Mol. Sci..

[73] Eichlerová I (2015). Enzymatic systems involved in decomposition reflects the ecology and taxonomy of saprotrophic fungi. Fungal Ecol..

[74] DiLegge MJ, Manter DK, Vivanco JM (2019). A novel approach to determine generalist nematophagous microbes reveals *Mortierella*
*globalpina* as a new biocontrol agent against *Meloidogyne* spp. nematodes. Sci. Rep..

[75] Huang X, Zhang N, Yong X, Yang X, Shen Q (2012). Biocontrol of *Rhizoctonia solani* damping-off disease in cucumber with *Bacillus*
*pumilus* SQR-N43. Microbiol. Res..

[76] Cheng X (2018). Characterization of antagonistic *Bacillus*
*methylotrophicus* isolated from rhizosphere and its biocontrol effects on maize stalk rot. Phytopathology.

[77] Smi A (2020). Biological control of tomato bacterial wilt by oxydifficidin and difficidin-producing *Bacillus*
*methylotrophicus* DR-08. Pestic. Biochem. Physiol..

[78] Ji C (2020). Effects of Bacillus methylotrophicus M4-1 on physiological and biochemical traits of wheat under salinity stress. J. Appl. Microbiol..

[79] Ahmad Z, Wu J, Chen L, Dong W (2017). Isolated *Bacillus*
*subtilis* strain 330-2 and its antagonistic genes identified by the removing PCR. Sci. Rep..

[80] Saha M (2016). Microbial siderophores and their potential applications: A review. Environ. Sci. Pollut. Res..

[81] Saeed Q (2021). Rhizosphere bacteria in plant growth promotion, biocontrol, and bioremediation of contaminated sites a comprehensive review of effects and mechanisms. Int. J. Mol. Sci..

[82] Djordjevic D, Wiedmann M, Mclandsborough LA (2002). Microtiter plate assay for assessment of *Listeria*
*monocytogenes* biofilm formation. Appl. Environ. Microbiol..

[83] Ji C, Liu Z, Hao L, Song X, Liu X (2020). Effects of *Enterobacter*
*cloacae* HG-1 on the nitrogen-fixing community structure of wheat rhizosphere soil and on salt tolerance. Front. Plant Sci..

[84] Smalla K, Cresswell N, Mendonca-Hagler LC, Wolters A, Elsas J (1993). Rapid DNA extraction protocol from soil for polymerase chain reaction-mediated amplification. J. Appl. Bacteriol..

[85] Olsen SR (1954). Estimation of Available Phosphorus in Soils by Extraction with Sodium Bicarbonate.

[86] Jackson, M. L., Jackson, M., Jackson, M. & Jackson, M. Soils chemical analysis: Advanced course (1956).

[87] Walkley AJ, Black IA (1934). An examination of the Degtjareff method for determining soil organic matter, and a proposed modification of the chromic acid titration method. Soil Sci..

[88] Guan, S. Y., Zhang, D. & Zhang, Z. *Soil Enzyme and Its Research Methods* 274–297 (1986).

[89] Yin R, Deng H, Wang HL, Zhang B (2014). Vegetation type affects soil enzyme activities and microbial functional diversity following re-vegetation of a severely eroded red soil in sub-tropical China. Catena.

[90] Tabatabai MA, Bremner JM (1969). Use of p-nitrophenyl phosphate for assay of soil phosphatase activity. Soil Biol. Biochem..

[91] Li Q (2014). Effect of land use on soil enzyme activities at karst area in Nanchuan, Chongqing, Southwest China. Plant Soil Environ..

