# Supplementary material for: Combined application of microbial inoculant and kelp-soaking wastewater promotes wheat seedlings growth and improves structural diversity of rhizosphere microbial community
Source: Sci Rep. 2023 Nov 24;13:20697. doi: 10.1038/s41598-023-48195-1 (PMC10673839; doi:10.1038/s41598-023-48195-1)
Supplement: Supplementary file 1 — Supplementary Information. [file 41598_2023_48195_MOESM1_ESM.pdf]

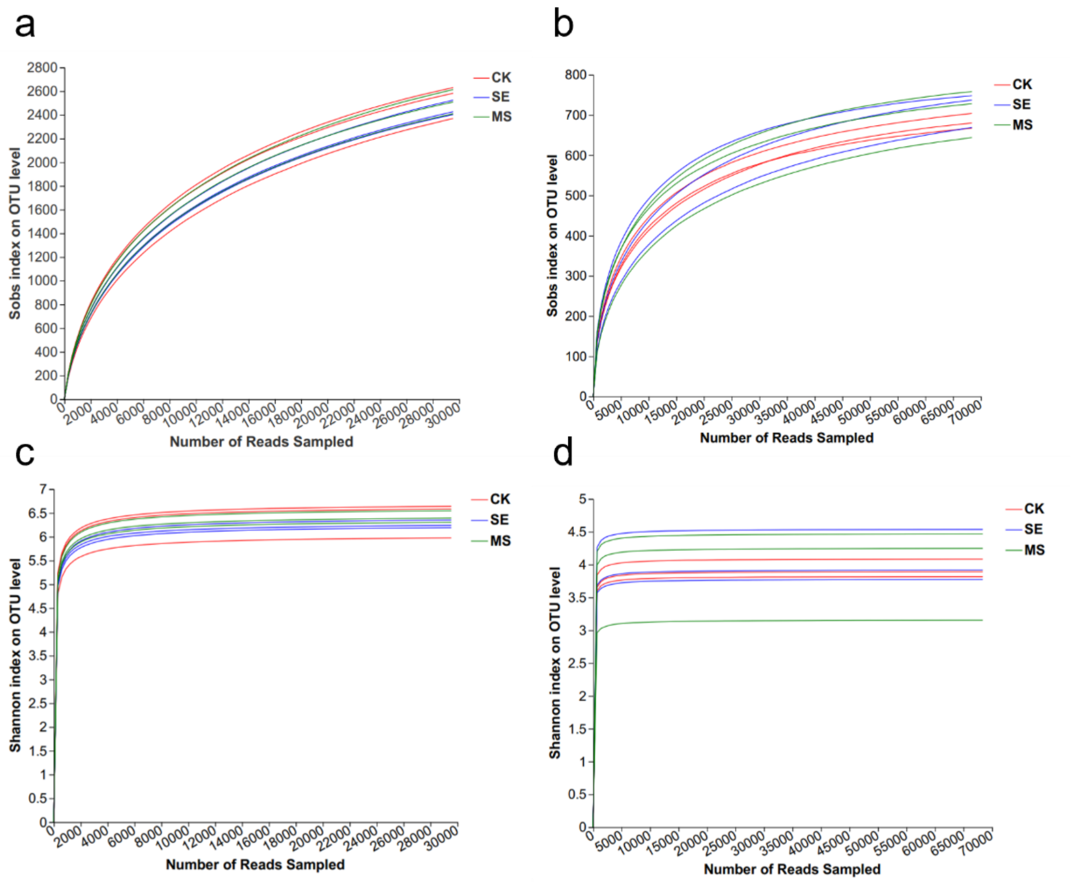

Figure S1 Rarefaction curves and Shannon curve

(A) Rarefaction curves of bacteria; (B) Rarefaction curves of fungi; (C) Shannon curves of bacteria; (D) Shannon curves of fungi.

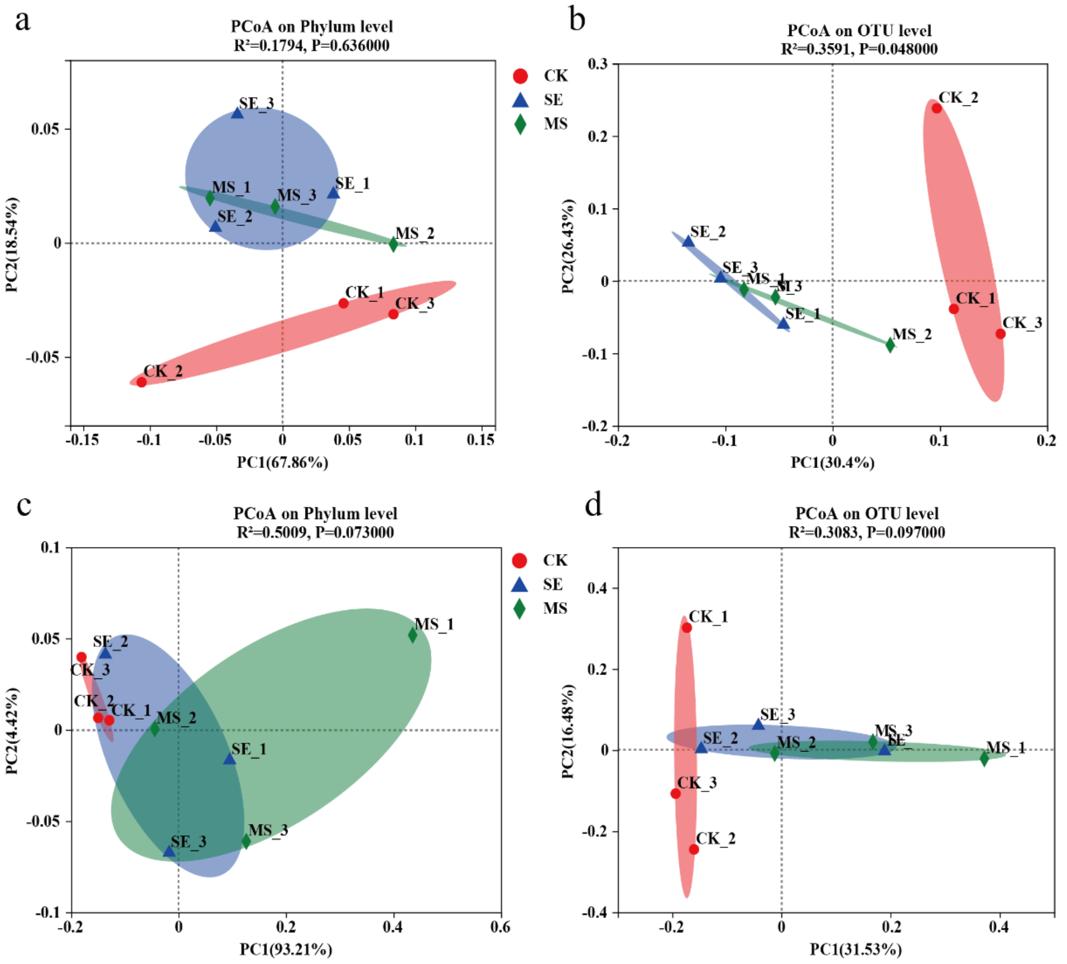

Figure S2 The principal co-ordinates analysis (PCoA) analysis based on Bray-Curtis distance was used to study the community composition of bacteria (a)(b) and fungus (c)(d) at phylum and OTU level.

Table S1 Environmental factors

Index	Bacteria		Fungi	
	r^2	p_values	r^2	p_values
AP	0.8908	0.4544	0.8473	0.005
OC	0.9915	0.1303	0.7481	0.011
AN	-0.5885	0.8085	0.5408	0.081
AK	0.9338	0.3578	0.8269	0.009
pH	0.9224	0.3862	0.8342	0.009
Urease	0.9954	-0.0959	0.6686	0.021
Phosphatase	0.9906	-0.1366	0.6561	0.023
Sucrase	-0.8817	-0.4718	0.8494	0.005
